# Poly[[μ_2_-acetato-aquadi-μ_3_-isonicotinato-erbium(III)silver(I)] perchlorate]

**DOI:** 10.1107/S1600536809048429

**Published:** 2009-11-21

**Authors:** Li-Cai Zhu

**Affiliations:** a School of Chemistry and Environment, South China Normal University, Guangzhou 510631, People’s Republic of China

## Abstract

In the title three-dimensional heterometallic complex, {[AgEr(C_6_H_4_NO_2_)_2_(C_2_H_3_O_2_)(H_2_O)]ClO_4_}_*n*_, the eight-coordin­ate Er^III^ ion adopts a distorted bicapped trigonal-prismatic geometry, being coordinated by four O atoms from four different isonicotinate ligands, three O atoms from two different acetate ligands and one O atom of the water mol­ecule. The two-coordinate Ag^I^ ion is surrounded by two N atoms from two different isonicotinate anions in a slightly bent configuration. These building blocks are connected by bridging isonicotinate and acetate ligands, generating a three-dimensional network. Ths structure is consolidated by O—H⋯O hydrogen bonding between the coordinated water mol­ecule and a carboxyl­ate group of the acetate ligand. The perchlorate anion is disordered over two sites with site-occupancy factors of 0.526 (13) and 0.474 (13), while the methyl group of the acetate ligand is equally disordered over two sites.

## Related literature

For background to lanthanide–transition metal heterometallic complexes, see: Cheng *et al.* (2006 ▶); Kuang *et al.* (2007 ▶); Peng *et al.* (2008 ▶); Zhu *et al.* (2009 ▶).
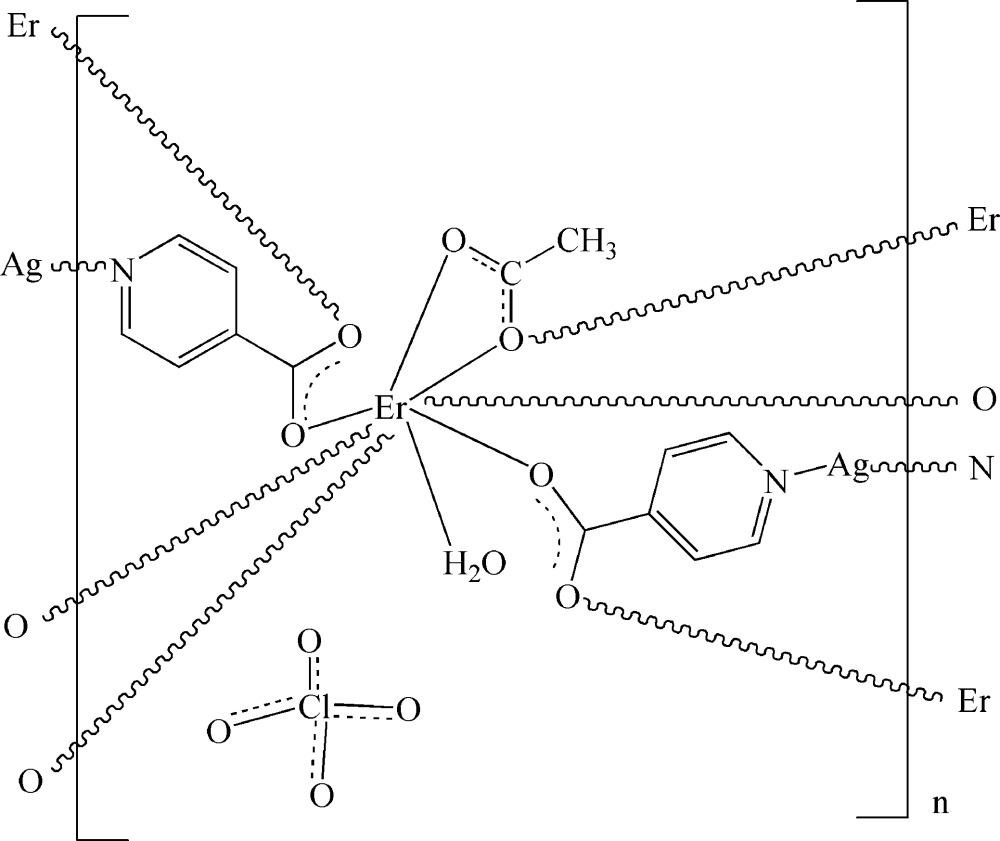


## Experimental

### 

#### Crystal data

[AgEr(C_6_H_4_NO_2_)_2_(C_2_H_3_O_2_)(H_2_O)]ClO_4_
*M* *_r_* = 695.84
Monoclinic, 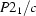

*a* = 16.1952 (11) Å
*b* = 14.8673 (11) Å
*c* = 7.8938 (6) Å
β = 91.783 (1)°
*V* = 1899.7 (2) Å^3^
*Z* = 4
Mo *K*α radiation
μ = 5.62 mm^−1^
*T* = 296 K
0.23 × 0.20 × 0.19 mm

#### Data collection

Bruker APEXII area-detector diffractometer
Absorption correction: multi-scan (**SADABS**; Sheldrick, 1996 ▶) *T*_min_ = 0.285, *T*_max_ = 0.344
9611 measured reflections
3423 independent reflections
3009 reflections with *I* > 2σ(*I*)
*R*_int_ = 0.030

#### Refinement

*R*[*F* ^2^ > 2σ(*F* ^2^)] = 0.025
*wR*(*F* ^2^) = 0.057
*S* = 1.05
3423 reflections
320 parameters
158 restraints
H atoms treated by a mixture of independent and constrained refinement
Δρ_max_ = 0.76 e Å^−3^
Δρ_min_ = −1.04 e Å^−3^

### 

Data collection: *APEX2* (Bruker, 2004 ▶); cell refinement: *SAINT* (Bruker, 2004 ▶); data reduction: *SAINT*; program(s) used to solve structure: *SHELXS97* (Sheldrick, 2008 ▶); program(s) used to refine structure: *SHELXL97* (Sheldrick, 2008 ▶); molecular graphics: *XP* in *SHELXTL* (Sheldrick, 2008 ▶); software used to prepare material for publication: *SHELXL97*.

## Supplementary Material

Crystal structure: contains datablocks I, global. DOI: 10.1107/S1600536809048429/wm2280sup1.cif

Structure factors: contains datablocks I. DOI: 10.1107/S1600536809048429/wm2280Isup2.hkl

Additional supplementary materials:  crystallographic information; 3D view; checkCIF report

## Figures and Tables

**Table 1:** Hydrogen-bond geometry (Å, °)

*D*—H⋯*A*	*D*—H	H⋯*A*	*D*⋯*A*	*D*—H⋯*A*
O1*W*—H2*W*⋯O4^i^	0.82 (4)	2.19 (3)	2.891 (5)	145 (5)
O1*W*—H1*W*⋯O6^ii^	0.81 (4)	1.99 (4)	2.786 (5)	167 (6)

Symmetry codes: (i) 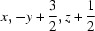; (ii) 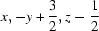.

**Fig. 1.**
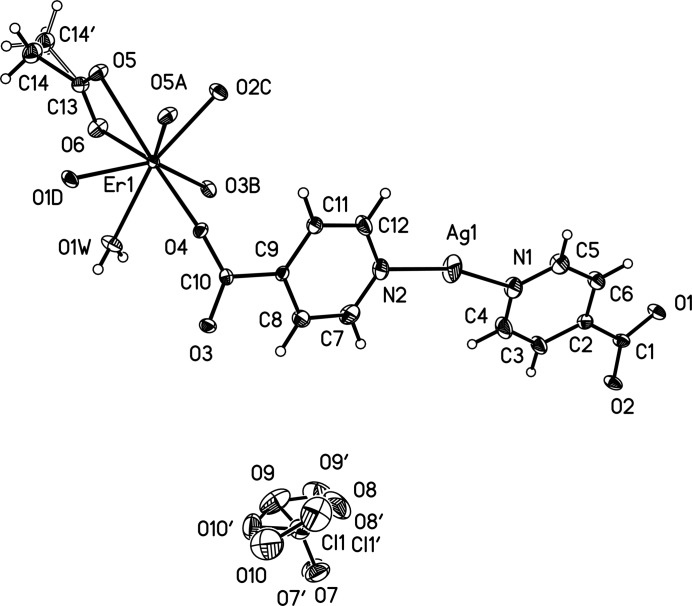
The molecular structure showing the atomic-numbering scheme and displacement ellipsoids drawn at the 30% probability level. Both disorder components are shown. Symmetry codes: (A) 2 - x, 2 - y, -z; (B) x, 1.5 - y, 1/2 + z; (C) 1 - x, 1/2 + y, 0.5 - z; (D) 1 + x, 1.5 - y, -1/2 + z.

**Fig. 2.**
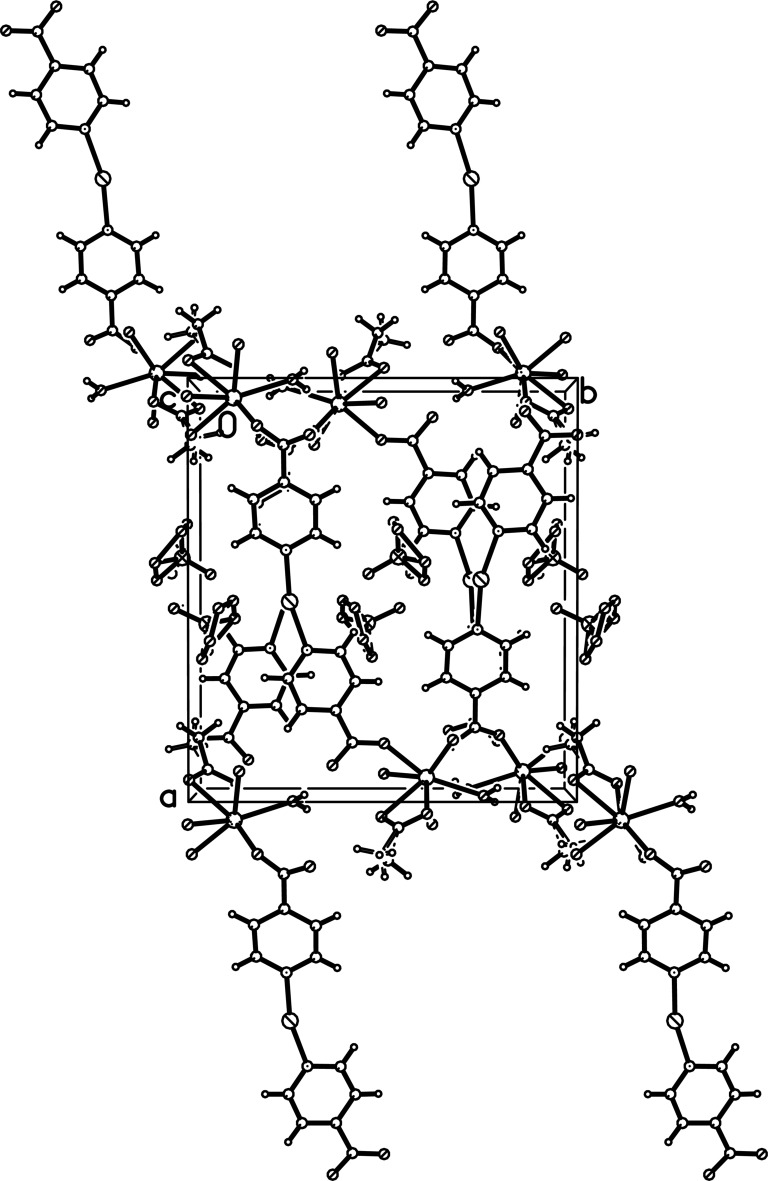
A view of the three-dimensional arrangement of the title compound. Hydrogen atoms were omitted for clarity.

## supplementary crystallographic information

### Comment

In the past few years, lanthanide-transition metal heterometallic complexes with bridging multifunctional organic ligands gained increasing interest, not only because of their crystal structures, but also due to their applications in ion exchange, magnetism, catalysis and as luminescent material (Cheng *et al.*, 2006; Kuang *et al.*, 2007; Peng *et al.*, 2008; Zhu *et al.*, 2009). As an extension of this research, the structure of the title compound, a new heterometallic coordination polymer, (I), has been determined and is presented in this article.

In the title compound (Fig. 1), there are one Er^III^ ion, one Ag^I^ ion, two halves of an acetate ligand, two isonicotinate ligands, one coordinated water molecule, and one perchlorate anion in the asymmetric unit. Each Er^III^ ion is eight-coordinated by four O atoms from four different isonicotinate ligands [Er—O distances ranging from 2.266 (3) to 2.308 (3) Å], three O atoms from two different acetate ligands [Er—O distances ranging from 2.341 (3) to 2.454 (3) Å], and one O atom of water molecule [Er—O distance 2.369 (3) Å]. The Er center can be described as having a distorted bicapped trigonal-prismatic coordination geometry. The two-coordinated Ag^I^ ion is bonded to two N atoms from two different isonicotinate anions [Ag—N distances 2.153 (4) and 2.154 (4) Å] and adopts a slight distortion from linearity with an N1—Ag1—N2 angle of 165.44 (19) °. These metal coordination units are connected by bridging isonicotinate and acetate ligands, generating a three-dimensional network (Fig. 2). The coordinated water molecules exhibit O—H···O hydrogen bonding to the uncoordinated O atom of a carboxylate group and to the acetate ligand (Table 1).

### Experimental

A mixture of AgNO_3_ (0.057 g, 0.33 mmol), Er_2_O_3_ (0.116 g, 0.33 mmol), isonicotinic acid (0.164 g, 1.33 mmol), CH_3_COONa (0.057 g, 0.7 mmol), H_2_O (7 ml), and HClO_4_ (0.257 mmol) (pH 2) was sealed in a 20 ml Teflon-lined reaction vessel at 443 K for 6 days and then slowly cooled to room temperature. The product was collected by filtration, washed with water and was air-dried. Colorless block-shaped crystals suitable for X-ray analysis were obtained.

### Refinement

H atoms bonded to C atoms were positioned geometrically and refined as riding, with C—H = 0.93 or 0.96 Å and *U*_iso_(H) = 1.2 or 1.5 *U*_eq_(C). H atoms of water molecules were found from difference Fourier maps and refined isotropically with a restraint of O—H = 0.81 - 0.82 Å. The perchlorate anion is disordered over two sites with site occupancy factors 0.526 (13) and 0.474 (13), whereas the methyl group of the acetate ligand is disordered over two sites with site occupancy factors 0.51 (5) and 0.49 (5).

### Crystal data

**Table:**

[AgEr(C_6_H_4_NO_2_)_2_(C_2_H_3_O_2_)(H_2_O)]ClO_4_	*F*(000) = 1324
*M**_r_* = 695.84	*D*_x_ = 2.433 Mg m^−^^3^
Monoclinic, *P*2_1_/*c*	Mo *K*α radiation, λ = 0.71073 Å
Hall symbol: -P 2ybc	Cell parameters from 4797 reflections
*a* = 16.1952 (11) Å	θ = 2.5–27.9°
*b* = 14.8673 (11) Å	µ = 5.62 mm^−^^1^
*c* = 7.8938 (6) Å	*T* = 296 K
β = 91.783 (1)°	Block, colourless
*V* = 1899.7 (2) Å^3^	0.23 × 0.20 × 0.19 mm
*Z* = 4	

### Data collection

**Table:**

Bruker APEXII area-detector diffractometer	3423 independent reflections
Radiation source: fine-focus sealed tube	3009 reflections with *I* > 2σ(*I*)
graphite	*R*_int_ = 0.030
φ and ω scan	θ_max_ = 25.2°, θ_min_ = 1.9°
Absorption correction: multi-scan (*SADABS*; Sheldrick, 1996)	*h* = −19→19
*T*_min_ = 0.285, *T*_max_ = 0.344	*k* = −17→17
9611 measured reflections	*l* = −5→9

### Refinement

**Table:**

Refinement on *F*^2^	Primary atom site location: structure-invariant direct methods
Least-squares matrix: full	Secondary atom site location: difference Fourier map
*R*[*F*^2^ > 2σ(*F*^2^)] = 0.025	Hydrogen site location: inferred from neighbouring sites
*wR*(*F*^2^) = 0.057	H atoms treated by a mixture of independent and constrained refinement
*S* = 1.05	*w* = 1/[σ^2^(*F*_o_^2^) + (0.0218*P*)^2^ + 3.7155*P*] where *P* = (*F*_o_^2^ + 2*F*_c_^2^)/3
3423 reflections	(Δ/σ)_max_ = 0.002
320 parameters	Δρ_max_ = 0.76 e Å^−^^3^
158 restraints	Δρ_min_ = −1.04 e Å^−^^3^

### Special details

**Table:**

Geometry. All e.s.d.'s (except the e.s.d. in the dihedral angle between two l.s. planes) are estimated using the full covariance matrix. The cell e.s.d.'s are taken into account individually in the estimation of e.s.d.'s in distances, angles and torsion angles; correlations between e.s.d.'s in cell parameters are only used when they are defined by crystal symmetry. An approximate (isotropic) treatment of cell e.s.d.'s is used for estimating e.s.d.'s involving l.s. planes.
Refinement. Refinement of *F*^2^ against ALL reflections. The weighted *R*-factor *wR* and goodness of fit *S* are based on *F*^2^, conventional *R*-factors *R* are based on *F*, with *F* set to zero for negative *F*^2^. The threshold expression of *F*^2^ > σ(*F*^2^) is used only for calculating *R*-factors(gt) *etc*. and is not relevant to the choice of reflections for refinement. *R*-factors based on *F*^2^ are statistically about twice as large as those based on *F*, and *R*- factors based on ALL data will be even larger.

### Fractional atomic coordinates and isotropic or equivalent isotropic displacement parameters (Å^2^)

**Table:**

	*x*	*y*	*z*	*U*_iso_*/*U*_eq_	Occ. (<1)
Er1	0.955305 (12)	0.883792 (13)	0.05025 (3)	0.01689 (8)	
Ag1	0.47352 (3)	0.74056 (4)	0.10055 (8)	0.06366 (18)	
N1	0.3573 (3)	0.6969 (3)	0.1973 (7)	0.0464 (13)	
N2	0.5953 (3)	0.7481 (3)	−0.0007 (6)	0.0378 (11)	
C1	0.1321 (3)	0.5802 (3)	0.3629 (7)	0.0287 (12)	
C2	0.2108 (3)	0.6229 (3)	0.3048 (7)	0.0273 (11)	
C3	0.2810 (3)	0.5718 (4)	0.2968 (8)	0.0427 (15)	
H8	0.2800	0.5114	0.3271	0.051*	
C4	0.3527 (4)	0.6105 (4)	0.2436 (9)	0.0523 (18)	
H7	0.4000	0.5753	0.2397	0.063*	
C5	0.2890 (3)	0.7466 (4)	0.2066 (9)	0.0455 (16)	
H11	0.2914	0.8070	0.1767	0.055*	
C6	0.2147 (3)	0.7121 (4)	0.2589 (8)	0.0378 (14)	
H10	0.1681	0.7485	0.2630	0.045*	
C7	0.6342 (3)	0.6716 (4)	−0.0395 (8)	0.0438 (15)	
H4	0.6076	0.6171	−0.0221	0.053*	
C8	0.7122 (3)	0.6709 (4)	−0.1041 (7)	0.0340 (13)	
H3	0.7372	0.6167	−0.1315	0.041*	
C9	0.7531 (3)	0.7512 (3)	−0.1281 (6)	0.0199 (10)	
C10	0.8387 (3)	0.7511 (3)	−0.1972 (6)	0.0203 (10)	
C11	0.7136 (3)	0.8299 (3)	−0.0871 (7)	0.0309 (12)	
H6	0.7394	0.8851	−0.1018	0.037*	
C12	0.6349 (3)	0.8258 (4)	−0.0237 (7)	0.0359 (13)	
H5	0.6085	0.8792	0.0040	0.043*	
O1	0.0753 (2)	0.6328 (2)	0.4074 (5)	0.0354 (9)	
O2	0.1298 (2)	0.4963 (2)	0.3618 (5)	0.0369 (9)	
O3	0.85978 (19)	0.6842 (2)	−0.2806 (4)	0.0276 (8)	
O4	0.88366 (18)	0.8187 (2)	−0.1684 (4)	0.0237 (7)	
O1W	0.9992 (2)	0.7323 (2)	0.0767 (5)	0.0327 (9)	
H1W	1.017 (3)	0.702 (3)	0.001 (5)	0.049*	
H2W	0.978 (3)	0.697 (3)	0.142 (5)	0.049*	
O6	1.0346 (2)	0.8847 (2)	0.3130 (4)	0.0324 (8)	
O5	1.0476 (2)	1.0034 (2)	0.1567 (4)	0.0289 (8)	
C13	1.0687 (3)	0.9591 (3)	0.2875 (6)	0.0266 (11)	
C14	1.1431 (13)	0.9863 (19)	0.395 (3)	0.040 (4)	0.51 (5)
H14A	1.1728	1.0327	0.3385	0.059*	0.51 (5)
H14B	1.1785	0.9351	0.4129	0.059*	0.51 (5)
H14C	1.1255	1.0082	0.5024	0.059*	0.51 (5)
C14'	1.1264 (15)	1.0028 (19)	0.417 (3)	0.040 (4)	0.49 (5)
H14D	1.1142	1.0659	0.4230	0.059*	0.49 (5)
H14E	1.1824	0.9947	0.3836	0.059*	0.49 (5)
H14F	1.1191	0.9756	0.5255	0.059*	0.49 (5)
Cl1	0.58069 (12)	0.04095 (12)	0.7436 (3)	0.0642 (5)	0.526 (13)
O7	0.5433 (10)	−0.0457 (8)	0.742 (2)	0.076 (5)	0.526 (13)
O8	0.5249 (9)	0.1082 (9)	0.709 (2)	0.133 (6)	0.526 (13)
O9	0.6203 (10)	0.0528 (9)	0.9021 (14)	0.109 (6)	0.526 (13)
O10	0.6454 (9)	0.0386 (10)	0.6206 (19)	0.134 (7)	0.526 (13)
Cl1'	0.58069 (12)	0.04095 (12)	0.7436 (3)	0.0642 (5)	0.474 (13)
O7'	0.5399 (11)	−0.0369 (9)	0.691 (2)	0.083 (7)	0.474 (13)
O8'	0.5676 (11)	0.1101 (10)	0.6227 (19)	0.124 (7)	0.474 (13)
O9'	0.5422 (11)	0.0751 (11)	0.8943 (18)	0.134 (7)	0.474 (13)
O10'	0.6643 (6)	0.0289 (8)	0.778 (2)	0.092 (5)	0.474 (13)

### Atomic displacement parameters (Å^2^)

**Table:**

	*U*^11^	*U*^22^	*U*^33^	*U*^12^	*U*^13^	*U*^23^
Er1	0.01467 (12)	0.01458 (12)	0.02167 (13)	−0.00074 (8)	0.00453 (8)	0.00002 (8)
Ag1	0.0248 (2)	0.0863 (4)	0.0813 (4)	−0.0136 (2)	0.0248 (3)	0.0023 (3)
N1	0.025 (2)	0.047 (3)	0.067 (4)	−0.007 (2)	0.017 (2)	0.000 (3)
N2	0.021 (2)	0.045 (3)	0.048 (3)	−0.003 (2)	0.013 (2)	0.000 (2)
C1	0.028 (3)	0.020 (3)	0.039 (3)	−0.005 (2)	0.012 (2)	−0.003 (2)
C2	0.024 (3)	0.028 (3)	0.031 (3)	−0.005 (2)	0.009 (2)	−0.003 (2)
C3	0.025 (3)	0.029 (3)	0.075 (5)	0.002 (2)	0.015 (3)	0.001 (3)
C4	0.026 (3)	0.048 (4)	0.084 (5)	0.004 (3)	0.017 (3)	0.000 (3)
C5	0.031 (3)	0.036 (3)	0.071 (5)	−0.009 (3)	0.016 (3)	0.009 (3)
C6	0.027 (3)	0.030 (3)	0.058 (4)	−0.001 (2)	0.015 (3)	0.005 (3)
C7	0.035 (3)	0.036 (3)	0.061 (4)	−0.016 (3)	0.012 (3)	0.002 (3)
C8	0.029 (3)	0.026 (3)	0.048 (4)	−0.003 (2)	0.012 (3)	−0.001 (2)
C9	0.016 (2)	0.023 (3)	0.020 (3)	−0.0031 (19)	0.0016 (19)	−0.0025 (19)
C10	0.018 (2)	0.023 (3)	0.021 (3)	0.0015 (19)	0.0023 (19)	0.002 (2)
C11	0.021 (2)	0.026 (3)	0.046 (3)	−0.004 (2)	0.010 (2)	−0.001 (2)
C12	0.026 (3)	0.035 (3)	0.047 (4)	0.007 (2)	0.012 (3)	0.002 (3)
O1	0.0258 (19)	0.0208 (19)	0.061 (3)	−0.0007 (15)	0.0249 (19)	0.0012 (16)
O2	0.0296 (19)	0.0196 (19)	0.063 (3)	−0.0016 (15)	0.0227 (18)	−0.0038 (17)
O3	0.0246 (17)	0.0214 (18)	0.037 (2)	−0.0021 (14)	0.0110 (16)	−0.0082 (15)
O4	0.0179 (16)	0.0250 (18)	0.0283 (19)	−0.0051 (14)	0.0042 (14)	−0.0058 (14)
O1W	0.045 (2)	0.0204 (18)	0.034 (2)	0.0068 (16)	0.0164 (18)	0.0022 (15)
O6	0.039 (2)	0.033 (2)	0.025 (2)	−0.0065 (16)	−0.0043 (16)	0.0089 (15)
O5	0.0335 (19)	0.0248 (18)	0.028 (2)	−0.0076 (15)	−0.0089 (16)	0.0043 (15)
C13	0.030 (3)	0.023 (3)	0.027 (3)	−0.001 (2)	−0.001 (2)	0.000 (2)
C14	0.038 (6)	0.042 (6)	0.037 (5)	−0.003 (5)	−0.011 (5)	0.000 (4)
C14'	0.038 (6)	0.042 (6)	0.037 (5)	−0.003 (5)	−0.011 (5)	0.000 (4)
Cl1	0.0638 (11)	0.0528 (10)	0.0756 (13)	0.0017 (9)	−0.0046 (10)	−0.0013 (9)
O7	0.071 (8)	0.070 (8)	0.089 (9)	−0.025 (6)	0.001 (6)	−0.009 (6)
O8	0.140 (10)	0.104 (9)	0.153 (11)	0.074 (7)	−0.032 (8)	−0.019 (7)
O9	0.134 (10)	0.110 (8)	0.081 (8)	−0.042 (7)	−0.036 (7)	0.001 (6)
O10	0.133 (10)	0.143 (10)	0.130 (10)	−0.033 (8)	0.052 (8)	−0.008 (8)
Cl1'	0.0638 (11)	0.0528 (10)	0.0756 (13)	0.0017 (9)	−0.0046 (10)	−0.0013 (9)
O7'	0.073 (9)	0.081 (9)	0.096 (10)	−0.033 (7)	0.011 (7)	−0.034 (7)
O8'	0.137 (11)	0.121 (10)	0.113 (10)	−0.009 (8)	−0.012 (8)	0.056 (8)
O9'	0.150 (11)	0.138 (10)	0.118 (10)	0.017 (8)	0.064 (8)	−0.032 (8)
O10'	0.054 (6)	0.077 (7)	0.144 (11)	0.004 (5)	−0.023 (7)	0.009 (7)

### Geometric parameters (Å, °)

**Table:**

Er1—O4	2.266 (3)	C11—C12	1.386 (7)
Er1—O2^i^	2.289 (3)	C11—H6	0.9300
Er1—O1^ii^	2.290 (3)	C12—H5	0.9300
Er1—O3^iii^	2.308 (3)	O1—Er1^v^	2.290 (3)
Er1—O5^iv^	2.341 (3)	O2—Er1^vi^	2.289 (3)
Er1—O1W	2.369 (3)	O3—Er1^vii^	2.308 (3)
Er1—O6	2.405 (3)	O1W—H1W	0.81 (4)
Er1—O5	2.454 (3)	O1W—H2W	0.82 (4)
Ag1—N1	2.153 (4)	O6—C13	1.256 (6)
Ag1—N2	2.154 (4)	O5—C13	1.263 (6)
N1—C5	1.336 (7)	O5—Er1^iv^	2.341 (3)
N1—C4	1.337 (8)	C13—C14	1.507 (9)
N2—C12	1.335 (7)	C13—C14'	1.507 (10)
N2—C7	1.341 (7)	C14—H14A	0.9600
C1—O2	1.248 (6)	C14—H14B	0.9600
C1—O1	1.266 (6)	C14—H14C	0.9600
C1—C2	1.507 (6)	C14'—H14D	0.9600
C2—C3	1.370 (7)	C14'—H14E	0.9600
C2—C6	1.376 (7)	C14'—H14F	0.9600
C3—C4	1.374 (8)	Cl1—O8	1.368 (9)
C3—H8	0.9300	Cl1—O10'	1.384 (8)
C4—H7	0.9300	Cl1—O7'	1.390 (9)
C5—C6	1.382 (7)	Cl1—O9	1.399 (9)
C5—H11	0.9300	Cl1—O8'	1.414 (10)
C6—H10	0.9300	Cl1—O7	1.423 (9)
C7—C8	1.376 (7)	Cl1—O10	1.451 (9)
C7—H4	0.9300	Cl1—O9'	1.452 (10)
C8—C9	1.381 (7)	O8—O8'	0.988 (19)
C8—H3	0.9300	O8—O9'	1.557 (19)
C9—C11	1.378 (7)	O9—O10'	1.278 (15)
C9—C10	1.505 (6)	O9—O9'	1.307 (17)
C10—O3	1.246 (5)	O10—O10'	1.280 (17)
C10—O4	1.258 (5)	O10—O8'	1.650 (18)
			
O4—Er1—O2^i^	104.11 (13)	O4—C10—C9	117.9 (4)
O4—Er1—O1^ii^	90.06 (13)	C9—C11—C12	119.1 (5)
O2^i^—Er1—O1^ii^	139.15 (12)	C9—C11—H6	120.4
O4—Er1—O3^iii^	85.24 (12)	C12—C11—H6	120.4
O2^i^—Er1—O3^iii^	73.96 (11)	N2—C12—C11	122.5 (5)
O1^ii^—Er1—O3^iii^	146.30 (12)	N2—C12—H5	118.7
O4—Er1—O5^iv^	77.04 (11)	C11—C12—H5	118.7
O2^i^—Er1—O5^iv^	71.89 (13)	C1—O1—Er1^v^	134.9 (3)
O1^ii^—Er1—O5^iv^	74.47 (12)	C1—O2—Er1^vi^	138.3 (3)
O3^iii^—Er1—O5^iv^	135.93 (12)	C10—O3—Er1^vii^	148.8 (3)
O4—Er1—O1W	78.85 (13)	C10—O4—Er1	139.3 (3)
O2^i^—Er1—O1W	148.19 (12)	Er1—O1W—H1W	125 (4)
O1^ii^—Er1—O1W	71.58 (12)	Er1—O1W—H2W	122 (4)
O3^iii^—Er1—O1W	74.77 (12)	H1W—O1W—H2W	106 (4)
O5^iv^—Er1—O1W	137.91 (12)	C13—O6—Er1	95.3 (3)
O4—Er1—O6	154.95 (12)	C13—O5—Er1^iv^	160.6 (3)
O2^i^—Er1—O6	92.59 (14)	C13—O5—Er1	92.8 (3)
O1^ii^—Er1—O6	89.25 (14)	Er1^iv^—O5—Er1	106.35 (13)
O3^iii^—Er1—O6	81.55 (12)	O6—C13—O5	118.7 (4)
O5^iv^—Er1—O6	126.62 (11)	O6—C13—C14	119.6 (11)
O1W—Er1—O6	77.18 (13)	O5—C13—C14	120.8 (11)
O4—Er1—O5	149.53 (11)	O6—C13—C14'	122.6 (12)
O2^i^—Er1—O5	74.51 (12)	O5—C13—C14'	118.3 (12)
O1^ii^—Er1—O5	74.32 (12)	O6—C13—Er1	58.3 (2)
O3^iii^—Er1—O5	122.10 (12)	O5—C13—Er1	60.6 (2)
O5^iv^—Er1—O5	73.65 (13)	C14—C13—Er1	167.2 (12)
O1W—Er1—O5	118.73 (13)	C14'—C13—Er1	177.2 (13)
O6—Er1—O5	52.98 (11)	C13—C14—H14A	109.5
O4—Er1—C13	169.94 (13)	C13—C14—H14B	109.5
O2^i^—Er1—C13	83.95 (14)	C13—C14—H14C	109.5
O1^ii^—Er1—C13	79.88 (14)	C13—C14'—H14D	109.5
O3^iii^—Er1—C13	102.99 (13)	C13—C14'—H14E	109.5
O5^iv^—Er1—C13	100.25 (13)	H14D—C14'—H14E	109.5
O1W—Er1—C13	97.55 (14)	C13—C14'—H14F	109.5
O6—Er1—C13	26.38 (13)	H14D—C14'—H14F	109.5
O5—Er1—C13	26.64 (12)	H14E—C14'—H14F	109.5
O4—Er1—Er1^iv^	114.47 (8)	O8—Cl1—O10'	140.3 (9)
O2^i^—Er1—Er1^iv^	68.88 (8)	O8—Cl1—O7'	104.2 (12)
O1^ii^—Er1—Er1^iv^	70.37 (8)	O10'—Cl1—O7'	113.8 (8)
O3^iii^—Er1—Er1^iv^	141.06 (8)	O8—Cl1—O9	111.5 (8)
O5^iv^—Er1—Er1^iv^	37.84 (8)	O10'—Cl1—O9	54.6 (7)
O1W—Er1—Er1^iv^	139.44 (9)	O7'—Cl1—O9	124.9 (10)
O6—Er1—Er1^iv^	88.78 (8)	O10'—Cl1—O8'	110.7 (8)
O5—Er1—Er1^iv^	35.81 (8)	O7'—Cl1—O8'	110.1 (9)
C13—Er1—Er1^iv^	62.42 (10)	O9—Cl1—O8'	124.5 (9)
N1—Ag1—N2	165.44 (19)	O8—Cl1—O7	112.4 (8)
C5—N1—C4	117.6 (5)	O10'—Cl1—O7	107.3 (10)
C5—N1—Ag1	126.0 (4)	O9—Cl1—O7	107.7 (8)
C4—N1—Ag1	116.3 (4)	O8'—Cl1—O7	126.7 (10)
C12—N2—C7	118.2 (4)	O8—Cl1—O10	111.8 (8)
C12—N2—Ag1	123.0 (4)	O10'—Cl1—O10	53.6 (7)
C7—N2—Ag1	118.8 (4)	O7'—Cl1—O10	97.3 (10)
O2—C1—O1	126.7 (4)	O9—Cl1—O10	106.3 (7)
O2—C1—C2	116.4 (4)	O8'—Cl1—O10	70.3 (8)
O1—C1—C2	116.9 (4)	O7—Cl1—O10	106.7 (8)
C3—C2—C6	118.5 (5)	O8—Cl1—O9'	66.9 (8)
C3—C2—C1	119.4 (5)	O10'—Cl1—O9'	109.0 (8)
C6—C2—C1	122.2 (5)	O7'—Cl1—O9'	108.9 (8)
C2—C3—C4	119.5 (5)	O9—Cl1—O9'	54.5 (7)
C2—C3—H8	120.2	O8'—Cl1—O9'	103.8 (8)
C4—C3—H8	120.2	O7—Cl1—O9'	97.5 (10)
N1—C4—C3	122.7 (5)	O10—Cl1—O9'	153.4 (9)
N1—C4—H7	118.6	O8'—O8—Cl1	71.7 (8)
C3—C4—H7	118.6	O8'—O8—O9'	123.3 (13)
N1—C5—C6	122.7 (5)	Cl1—O8—O9'	59.1 (6)
N1—C5—H11	118.6	O10'—O9—O9'	126.6 (10)
C6—C5—H11	118.6	O10'—O9—Cl1	62.1 (6)
C2—C6—C5	119.0 (5)	O9'—O9—Cl1	64.8 (7)
C2—C6—H10	120.5	O10'—O10—Cl1	60.5 (6)
C5—C6—H10	120.5	O10'—O10—O8'	102.8 (9)
N2—C7—C8	122.2 (5)	Cl1—O10—O8'	53.8 (5)
N2—C7—H4	118.9	O8—O8'—Cl1	66.8 (8)
C8—C7—H4	118.9	O8—O8'—O10	122.7 (11)
C7—C8—C9	119.7 (5)	Cl1—O8'—O10	55.9 (6)
C7—C8—H3	120.2	O9—O9'—Cl1	60.7 (5)
C9—C8—H3	120.2	O9—O9'—O8	105.7 (9)
C11—C9—C8	118.2 (4)	Cl1—O9'—O8	54.0 (5)
C11—C9—C10	121.7 (4)	O9—O10'—O10	126.2 (10)
C8—C9—C10	120.0 (4)	O9—O10'—Cl1	63.3 (6)
O3—C10—O4	124.5 (4)	O10—O10'—Cl1	65.9 (6)
O3—C10—C9	117.6 (4)		

Symmetry codes: (i) −*x*+1, *y*+1/2, −*z*+1/2; (ii) *x*+1, −*y*+3/2, *z*−1/2; (iii) *x*, −*y*+3/2, *z*+1/2; (iv) −*x*+2, −*y*+2, −*z*; (v) *x*−1, −*y*+3/2, *z*+1/2; (vi) −*x*+1, *y*−1/2, −*z*+1/2; (vii) *x*, −*y*+3/2, *z*−1/2.

### Hydrogen-bond geometry (Å, °)

**Table:**

*D*—H···*A*	*D*—H	H···*A*	*D*···*A*	*D*—H···*A*
O1W—H2W···O4^iii^	0.82 (4)	2.19 (3)	2.891 (5)	145 (5)
O1W—H1W···O6^vii^	0.81 (4)	1.99 (4)	2.786 (5)	167 (6)

Symmetry codes: (iii) *x*, −*y*+3/2, *z*+1/2; (vii) *x*, −*y*+3/2, *z*−1/2.
